# Age-Related Differences in the Modulation of Small-World Brain Networks during a Go/NoGo Task

**DOI:** 10.3389/fnagi.2016.00100

**Published:** 2016-05-17

**Authors:** Xiangfei Hong, Yuelu Liu, Junfeng Sun, Shanbao Tong

**Affiliations:** ^1^Shanghai Key Laboratory of Psychotic Disorders, Shanghai Mental Health Center, Shanghai Jiao Tong University School of MedicineShanghai, China; ^2^School of Biomedical Engineering, Shanghai Jiao Tong UniversityShanghai, China; ^3^Center for Mind and Brain, University of CaliforniaDavis, CA, USA

**Keywords:** aging, graph theory, induced activity, phase synchrony, response inhibition

## Abstract

Although inter-regional phase synchrony of neural oscillations has been proposed as a plausible mechanism for response control, little is known about the possible effects due to normal aging. We recorded multi-channel electroencephalography (EEG) from healthy younger and older adults in a Go/NoGo task, and examined the aging effects on synchronous brain networks with graph theoretical analysis. We found that in both age groups, brain networks in theta, alpha or beta band for either response execution (Go) or response inhibition (NoGo) condition showed prominent small-world property. Furthermore, small-world property of brain networks showed significant differences between different task conditions. Further analyses of node degree suggested that frontal-central theta band phase synchrony was enhanced during response inhibition, whereas during response execution, increased phase synchrony was observed in beta band over central-parietal regions. More interestingly, these task-related modulations on brain networks were well preserved and even more robust in older adults compared with younger adults. Taken together, our findings not only suggest that response control involves synchronous brain networks in functionally-distinct frequency bands, but also indicate an increase in the recruitment of brain network resources due to normal aging.

## Introduction

Response inhibition, the ability to inhibit a prepotent tendency of behavioral response, is a core component of human cognitive control functions (Diamond, 2013). Previous electroencephalography (EEG) studies regularly focused on the event-related potentials (ERPs) evoked by Go and NoGo (or Stop) stimuli in Go/NoGo (or Stop-Signal) response control paradigm. Two ERP components, i.e., N2 and P3, were consistently identified in the ERP waves elicited by the NoGo (or Stop) stimuli compared with the Go stimuli, typically observed over frontal-central cortex after ~200 ms and ~300 ms post-stimulus onset, respectively. Such ERP components were usually interpreted as the neural markers of response inhibition (Falkenstein et al., 1999; Albert et al., 2013; Huster et al., 2013), and were also shown to be sensitive to normal aging (Hong et al., 2014).

Besides the analysis of ERP, frequency or time-frequency domain analysis on oscillatory EEG power during Go/NoGo tasks has also been reported in several studies. For example, states or tasks that require an increased level of cognitive control, i.e., NoGo condition, always evoked higher power of theta oscillations during 200–600 ms post-stimulus period over the frontal-central scalp (Kirmizi-Alsan et al., 2006; Huster et al., 2013; Ergen et al., 2014). As a widely replicated finding, frontal theta activity was recently proposed to be a plausible mechanism for cognitive control functions (Cavanagh and Frank, 2014). While during movement execution tasks (Go condition), decreased power of alpha and beta oscillations over the sensorimotor areas was a common finding in the literature (Leocani et al., 1997; Pfurtscheller and Lopes da Silva, 1999).

Although ERP and frequency-domain analyses have been the dominating techniques in previous EEG research on Go/NoGo tasks, recent studies began to focus on the neural oscillations from the perspective of inter-regional phase synchrony in different frequency bands, and suggested an important role of large-scale neural synchrony in response inhibition (Serrien et al., 2005; Moore et al., 2008; Tallet et al., 2009; Brier et al., 2010; Muller and Anokhin, 2012; Anguera et al., 2013b; Cavanagh and Frank, 2014). For example, one recent study suggested that the effective inhibition of a prepotent response should be associated with an increase of the theta-band phase synchrony between the frontal and parietal cortices in a Go/NoGo task (Muller and Anokhin, 2012), and another study reported inhibition-specific changes in beta-band phase coherence between cerebral motor areas in a stop task (Tallet et al., 2009).

In spite of accumulating studies reporting the role of phase synchrony among healthy young adults, the influences of normal aging during an active inhibitory state have been scarce in the literature. Nonetheless, there have been phase synchrony studies that mainly focused on the pathological aging population, i.e., Alzheimer’s disease, showing the reduced phase synchrony during either the resting state (Uhlhaas and Singer, 2006; Stam et al., 2007, 2009; Knyazeva et al., 2010) or task states such as working memory (Pijnenburg et al., 2004) compared with normal elders. More interestingly, a recent study showed an increase of frontal-posterior theta-band phase coherence in healthy older adults following cognitive training that resulted in performance benefits (Anguera et al., 2013a). However, although such findings consistently implied a close relationship between cognitive control and neural synchrony during cognitive aging, a comprehensive and direct comparison of neural synchrony between healthy young and old adults from the perspective of large-scale neural synchrony is still lacking.

We inferred that there might be different possibilities on the results. On one hand, inspired by previous findings that the decline in cognitive ability was always accompanied with reduced neural synchrony during pathological aging (Uhlhaas and Singer, 2006), one may infer that the cognitive decline during normal aging would implicate a reduction in the ability to modulate neural synchrony for older adults compared with younger adults. Yet alternatively, from the perspective of compensatory mechanisms of cognitive aging, older adults might recruit additional brain activation to partially compensate the cognitive decline (Cabeza et al., 2002; Rajah and D’Esposito, 2005; Park and Reuter-Lorenz, 2009; Grady, 2012), which would lead to the enhancement of the neural synchrony in older adults. Nonetheless, in either case, investigating the effects of normal aging on neural synchrony during a cognitive control tasks would provide valuable insights for understanding the age-related changes in neural mechanisms of cognitive control functions.

In this study, we aimed to study the influence of normal aging on neural synchrony during response control. To this end, we recorded multi-channel EEG from healthy younger and older adults in a Go/NoGo response control task. Frequency-domain analysis was performed to examine task-related modulations on oscillatory EEG power, which could provide useful information for the comparison with existing literature. After that, functional brain networks were constructed based on phase synchrony analysis (Sun et al., 2012). Graph theory was then used to analyze the topological organizations of oscillatory brain networks during Go and NoGo conditions (Bullmore and Sporns, 2009; Rubinov and Sporns, 2010). We expected to observe the task-related modulations on brain networks between response execution (Go) and response inhibition (NoGo) conditions in functionally-distinct frequency bands, i.e., theta, alpha and beta bands (Tallet et al., 2009; Brier et al., 2010; Muller and Anokhin, 2012; Anguera et al., 2013b). Furthermore, we compared the task-modulated brain networks between younger and older adults to investigate age-related differences in neural synchrony during response inhibition and execution.

## Materials and Methods

### Participants

We recruited 23 healthy younger (mean ± standard deviation; age: 21.4 ± 2 years; range: 18–25 years; 7 females; all right-handed) and 18 healthy older adults (mean ± standard deviation; age: 61 ± 6 years; range: 50–70 years; 11 females; all right-handed) as participants. All participants reported normal or corrected-to-normal vision, without a history of neurological or psychiatric disorders. All older participants went through the Mini-Mental Status Examination (mean score: 28/30; range: ≥26; Folstein et al., 1975) with normal cognition. A minimum of 9 years of school education was required for each participant. All participants gave their written informed consents before the experiment, and were financially compensated for the experiment regardless of their performance. The experimental protocols were approved by the institutional Ethical Committee of Shanghai Jiao Tong University, complying with the Declaration of Helsinki.

### Stimuli and Procedures

A modified visual Go/NoGo task was adopted in this study. Before each trial, a black central crosshair (1.38° by 1.38°) and two lateral black location markers (2.39° by 2.39°, located 9.05° from the vertical meridian, and 7.2° below the horizontal meridian) were constantly presented on a white background. Participants were instructed to always maintain fixation on the crosshair whenever it was displayed. Each trial began with a 200 ms central black arrow cue pointing to either the left (50%) or the right (50%). Subjects were required to covertly attend the left or the right location according to the cue and ignore the other location. The target stimulus (1.67° by 1.67°), either a plus sign (50%) or the letter “x” (50%), was presented for 200 ms inside either the left (50%) or the right (50%) location marker after a random cue-target interval (CTI: jittered between 1000–1200 ms). Subjects were required to respond only to the plus sign presented in the attended location (Go trials) as quickly and accurately as possible with the right index finger, and refrain from responding to the letter “x” presented in the attended location marker (NoGo trials). Targets presented in the unattended location marker were to be ignored completely. A fixed inter-trial interval of 2600 ms was presented between the target offset and the cue onset of the next trial. For the Go trials, responses within 1600 ms after the target offset were recorded as valid trials.

Participants were comfortably seated in a sound attenuated room during the experiment. All stimuli were presented on a 19 inch LCD display (Dell: P190SB) placed 60 cm in front of the participant. The experimental paradigm was implemented in E-Prime (Version: 2.0, Psychology Software Tools, Inc., Sharpsburg, PA, USA), and behavioral responses were recorded with the Serial Response Box^TM^ included in the E-Prime toolkit. Each block consisted of 60 trials lasting for about 5 min. To minimize subject fatigue, a short (2–3 min) break was included between two successive blocks. All subjects went through a training block to get familiar with the experimental procedures. After the training, all younger subjects completed eight blocks of formal experiment trials and older subjects completed six blocks, considering the fact that the elders are more likely to develop mental fatigue that could affect the brain activity (Sun et al., 2014). To eliminate potential differences due to unequal trial numbers in the two groups, further EEG analyses only included data from the first six blocks in the younger group.

### EEG Recording and Preprocessing

Continuous EEG signals were recorded from 32 scalp electrodes (30 recording channels: Fp1, Fp2, F3, F4, F7, F8, Fz, FC1, FC2, FC5, FC6, C3, C4, Cz, T7, T8, CP1, CP2, CP5, CP6, P3, P4, P7, P8, 171 Pz, O1, O2, Oz, TP9, TP10; recording reference: FCz; ground: AFz) using the BrainAmp MR Plus amplifier and EasyCap^TM^ (Brain Products GmbH, Gilching, Germany). Two additional electrodes were placed on the outer left canthus and above the right eye to record horizontal electrooculogram (HEOG) and vertical electrooculogram (VEOG), respectively. EEG signals were amplified and sampled at 1000 Hz with 0.016–100 Hz online band-pass filtering. Impedance of each electrode was maintained below 10 kΩ during the recording.

EEG preprocessing was performed in the Matlab-based (MathWorks, MA, USA) EEGLAB (Delorme and Makeig, 2004) and ERPLAB toolboxes (Lopez-Calderon and Luck, 2014). Raw continuous EEG data first went through a two-way, zero phase shift, Butterworth filter (band-pass: 0.1–40 Hz; roll-off slope: 12 dB/oct), followed by a Parks McClellan notch filter to eliminate remaining noise at 50 Hz. Independent component analysis was performed to remove the ocular artifacts (Jung et al., 2000). Continuous EEG data were then re-referenced to the average of bilateral mastoid electrodes (TP9 and TP10), and segmented into epochs from −200 to 800 ms referring to the target onsets. Epochs with physical artifact in any EEG channel were marked as bad epochs according to the following criteria: (1) the maximal absolute value of voltage difference within a moving window (width: 200 ms; step: 50 ms) exceeding 150 μV; and (2) the maximal absolute value of voltage at any time point exceeding 100 μV. Furthermore, EEG epochs with overt eye movements or blinks that might prevent subjects from recognizing the targets were marked as bad epochs according to the following criteria: (1) the maximal absolute value of voltage difference in the HEOG channel within a moving window (width: 400 ms; step: 10 ms) exceeding 40 μV; and (2) the maximal absolute value of voltage difference in the VEOG channel at any time point around the target (−200 to 200 ms post-stimulus) exceeding 50 μV. After that, all EEG epochs were further visually inspected and all bad epochs were excluded in subsequent analyses.

In this study, the 200–700 ms post-stimulus period was chosen for the following analysis of EEG spectral power and phase synchrony, considering that: (1) our previous study has shown that this window covers the processes related to response inhibition, as suggested by the ERP components (N2 and P3) observed during this window (Hong et al., 2014); (2) from the computational perspective, our previous research based on surrogate tests has shown that an epoch of 500 ms yields optimal results for phase synchrony analysis in EEG theta, alpha and beta bands (Sun et al., 2012). For the sake of comparison, the last 500 ms of inter-trial interval (−500 to 0 pre-cue) was selected as the Baseline condition, which was included in the following analysis as reference.

**Figure 1:**
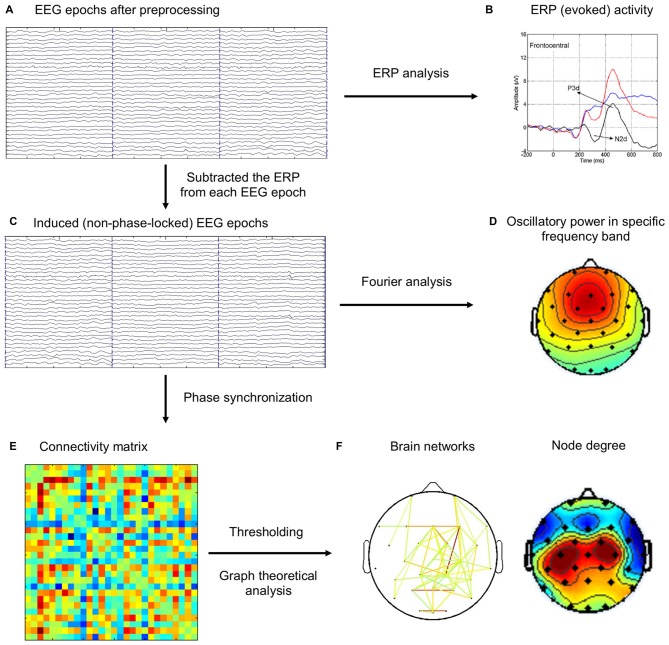
**The schematic diagram for the brain network analysis. (A)** Electroencephalography (EEG) epochs after preprocessing steps. **(B)** The event-related potentials (ERPs) were computed by averaging the same type of EEG epochs (Go, NoGo), which was reported in our previous study (Hong et al., 2014). **(C)** ERP activity was subtracted from EEG epochs. **(D)** Frequency-domain analysis of oscillatory power using Fourier analysis. **(E)** Phase synchronization (PS) index was computed for each pair of channels in each trial, and the connectivity matrix was averaged across the same type of EEG epochs (Baseline, Go, NoGo). **(F)** Different thresholds were applied on the connectivity matrix to construct brain networks, which were then analyzed using graph theoretical metrics.

It has been widely agreed that the event-related EEG includes both evoked and induced activities (Pfurtscheller and Lopes da Silva, 1999; Bastiaansen and Hagoort, 2003). The evoked activity, directly driven by the stimulus and both time- and phase-locked to it, can be extracted from the ongoing EEG by a straightforward averaging of EEG epochs, resulting in the ERP. The induced activity, on the other hand, is largely rhythmic (oscillatory) in nature, refers to oscillations caused or modulated by stimuli or state changes that do not directly drive the rhythm, so that they are time-locked, but not necessarily phase-locked, to the eliciting event. Moreover, previous studies have suggested to remove evoked activity when analyzing induced activity (Dietl et al., 1999; Doppelmayr et al., 2000; Gruber et al., 2002; Deiber et al., 2009). In this study, we are only interested in induced activity, because evoked activity (ERP, N2 and P3 components) has been analyzed and reported in our previous study (Hong et al., 2014). Therefore, ERP activity from each task condition (Go, NoGo) was subtracted from EEG epochs of the same condition for each subject to eliminate the contributions from evoked activity before subsequent analyses of induced activity. The flowchart of EEG analysis was illustrated in Figure 1.

### EEG Spectral Power Analysis

To analyze the modulation of EEG band power during the Go/NoGo task, we computed fast Fourier transform for the 200–700 ms post-stimulus period with Hamming window for each electrode, and then averaged the spectra across all trials of the same experimental condition (Baseline, NoGo, Go). To eliminate the inter-subject variance, task-related power change was computed as the percentage change of spectral power between different task conditions (Hong et al., 2013).

### Phase Synchronization Analysis

Phase synchronization (PS) has been successfully used to analyze rhythmic synchrony in oscillatory neural signals (Sun et al., 2012; Hong et al., 2013; Yan et al., 2013). The strength of phase synchrony can be quantified by PS index (PSI), which is based on the instantaneous phase (IP) of oscillations. For an epoch of real-value narrow-band EEG signal *s*(*t*), its IP can be defined as:

(1) $$\phi (t)=arg[z(t)]=arctan\frac{\tilde{s}(t)}{s(t)}\;(1)$$

where,

(2) $$z(t)=s(t)+j\tilde{s}(t)\;(2)$$

is the analytic signal of *s*(*t*), and

(3) $$\tilde{s}(t)=\frac{1}{\pi }\text{P}.\text{V}.\int_{-\infty }^{\infty }\frac{s(\tau )}{t-\tau }d\tau \;(3)$$

is the Hilbert transform of *s*(*t*) (P.V. denotes that the integral is taken in the sense of Cauchy principal value). Let *φ*_1_(*t*) and *φ*_2_(*t*) denote the IPs of two narrow-band EEG waves from two EEG channels during the same period. If the IP difference, i.e., |*mφ_1_(t) − nφ_2_(t)*|, is bounded by a constant, this pair of EEG waves are deemed to be in *m*:*n* PS, where *m* and *n* are positive integers (Tass et al., 1998; Wacker and Witte, 2011). In this study, we followed the approach adopted in recent studies of phase synchrony and only focused on the 1:1 PS (Sun et al., 2012; Hong et al., 2013; Yan et al., 2013). In this case, the PSI (*ρ*) can be quantified as the mean phase coherence of the IP difference, i.e.,

(4) $$\rho =\sqrt{{\langle \cos\left[\phi_{1}(t)-\phi_{2}(t)\right]\rangle }_{t}^{2}+{\langle \sin\left[\phi_{1}(t)-\phi_{2}(t)\right]\rangle }_{t}^{2}}\text{​​ }(4)$$

where 〈 ⋅ 〉 denotes the average over time. The value of PSI (*ρ*) is in the range of [0 1], with *ρ* = 0 indicating no PS at all and *ρ* = 1 indicating perfect PS.

In this study, the PS analysis for each EEG epoch was performed as follows: (1) filtering the EEG epochs into different frequency bands (theta: 4–8 Hz; alpha: 8–13 Hz; beta: 13–30 Hz); (2) computing the IP of the sub-band EEG signals according to Eq (1) for each epoch; (3) computing the PSI between each pair of electrodes using Eq (4); and (4) assigning the PSIs into an association matrix (28 × 28 in this study), with element in the i^th^ row and j^th^ column, *ρ_ij_*, representing the PSI between channel *i* and channel *j*. Finally, we averaged PSI matrices from all epochs under the same experimental conditions (Baseline, Go, NoGo) for each subject and obtained three PSI matrices (Baseline, Go, NoGo) in each frequency band for each subject.

### Graph Theoretical Analysis

In graph theoretical analysis, each channel is defined as a node, and the connectivity strength between two nodes is designated as the edge that connects them. The association matrix (*ρ_ij_*) was converted into a weighted graph (*w_ij_*) by applying a threshold (*T*) to eliminate those weak and spurious connections, i.e.,

(5) $$w_{ij}=\{\begin{array}{cc} \rho_{ij}, & \text{if}\geq T\\ 0, & \text{otherwise}. \end{array}\;(5)$$

where *w*_ij_ denotes the connectivity strength between node *i* and node *j*. The threshold value *T* was determined via a commonly used approach which explores the brain graph as a function of the changing threshold (Bullmore and Bassett, 2011; Hong et al., 2013; Yan et al., 2013). Previous studies have shown that the efficient organization of brain networks is typically observed in relatively sparse networks with network densities (the ratio between the existing edge number and maximal possible edge number) being less than 0.5, and that the maximal cost-efficiency of brain networks are typically reached at a network density of around 0.3 (Achard and Bullmore, 2007; Bassett et al., 2009; Bullmore and Bassett, 2011; Bullmore and Sporns, 2012; Jin et al., 2012). Therefore, in this study, we constructed connectivity graphs under a series of edge numbers (*K*) ranging from 60–180 with a step of 20. Specifically, for a given edge number *K*, the threshold (*T*) was assigned as the *K*^th^ largest value among all PSIs. The corresponding network density hence ranged approximately from 0.16 to 0.48.

The degree of a node, defined as the total connectivity strength of the corresponding node, was used to describe the importance of that node in the graph. Nodes with high degrees are regarded as hubs and are likely to play an important role in network communications (Bullmore and Sporns, 2009; Bullmore and Bassett, 2011). For weighted networks, the degree of node *i* (*D*_i_) is quantified as:

(6) $$D_{i}=\sum_{j\in N}w_{ij}\text{​ }(6)$$

where *N* denotes the set of all nodes in the network.

Recent research has shown that brain networks typically exhibit the so-called “small-world” property, which is thought to reflect an efficient organization with an optimal compromise between local segregation and global integration (Bassett et al., 2006; Achard and Bullmore, 2007; Jin et al., 2012). In this study, we will compare the small-world property of functional brain networks between young and old adults within different frequency bands to explore age-related reorganizations during the response inhibition task. Generally, small-world networks are characterized as networks with significantly greater local segregation but approximately the same level of global integration compared with random networks (Watts and Strogatz, 1998; Rubinov and Sporns, 2010; Bullmore and Sporns, 2012). Clustering coefficient is a measure indicating the level of local segregation of a network (Rubinov and Sporns, 2010). For a weighted network, the clustering coefficient is defined as the average clustering coefficient between all nodes in the network (Onnela et al., 2005),

(7) $$C=\;\frac{1}{n}\sum_{i\in N}C_{i}=\;\frac{1}{n}\sum_{i\in N}\left[\frac{1}{D_{i}(D_{i}-1)}\sum_{j,h\in N}{\left(w_{ij}w_{ih}w_{jh}\right)}^{1/3}\right]\;(7)$$

where *n* is number of nodes in the graph and *D*_i_ is the degree for node *i* as defined in Eq (6) Characteristic path length, on the other hand, describes the level of global integration of a network (Rubinov and Sporns, 2010). It is defined as the average shortest path length between all pairs of nodes (Latora and Marchiori, 2001),

(8) $$L=\frac{1}{\frac{1}{n(n-1)}\sum_{i\neq j\in N}\frac{1}{d_{ij}}}\;(8)$$

where *d*_ij_ denotes the shortest path length between node *i* and node *j*.

To examine the small-world property of functional brain networks, the clustering coefficient and characteristic path length were compared with those from 20 size-matched random networks generated from randomly rewiring the original brain networks (Maslov and Sneppen, 2002). This procedure yielded the normalized clustering coefficient γ = *C/C*_rand_ and characteristic path length λ = *L/L*_rand_, where *C*_rand_ and *L*_rand_ denote the average clustering coefficient and characteristic path length of the 20 random networks, respectively. The small-world property can then be quantified by the small-worldness index (Humphries and Gurney, 2008),

(9) $$\sigma =\frac{\gamma }{\lambda }\;(9)$$

For a typical small-world network, σ is greater than 1 (*γ > * 1, λ ≈ 1). Note that the graph theoretical analysis was performed in Matlab with Brain Connectivity Toolbox (Rubinov and Sporns, 2010).

### Statistical Analysis

For the analysis of *C* and *L*, we used the normalized values, i.e., *γ* and λ, to eliminate possible influences from connectivity strength (Rubinov et al., 2009), and performed the statistical analysis under different network density levels. It should be noted that: (1) the purpose of constructing brain graphs under a series of network density levels was to cover the *real* network density level that has been suggested to be located in the pre-defined range as much as possible; (2) the brain graphs under different network density levels are far from independent graphs, and thus the correction for multiple comparisons, i.e., Bonferroni correction, is not appropriate here (Stam et al., 2007; Rubinov et al., 2009; Jin et al., 2012; Hong et al., 2013; Li et al., 2015). Instead of the correction for *p*-values, in this study, we did not treat the results as significant unless the *p* < 0.05 significance level was observed under at least 3 (out of 7) different network density levels. For the statistical analysis of node degree, we chose a specific threshold around the median edge density of 0.3 (120 edges; network density: 120/378 = 0.3175) that is typically regarded as the most economical network density level (Achard and Bullmore, 2007; Bassett et al., 2009; Bullmore and Bassett, 2011). Statistical significance of network measures were assessed by the repeated-measures analysis of variance (ANOVA), independent-samples *t*-test and paired-samples *t*-test (two-tailed). Statistical analysis was performed in SPSS 16.0.

## Results

### Behavioral Performance

The behavioral results have been reported elsewhere (Hong et al., 2014). Briefly, the overall accuracy including both Go and NoGo trials was marginally higher for younger adults compared with older adults (younger: 99.52% ± 0.08% vs. older: 98.91% ± 0.31%; *t*_(18.027)_ = 1.893, *p* = 0.074). Older adults responded more slowly to Go targets than younger adults (younger: 477.56 ± 10.74 ms vs. older: 556.49 ± 28.46 ms; *t*_(20.582)_ = −2.595, *p* = 0.017). Furthermore, the analysis of false alarm rate (FAR) to NoGo-targets at the attended location showed no significant differences between the two groups (younger: 0.52% ± 0.13% vs. older: 1.17% ± 0.50%; *t*_(18.115)_ = −1.250, *p* > 0.2). Taken together, behavioral results suggested that although the response was slower due to aging, both younger and older adults showed satisfactory and comparable inhibitory performances in the Go/NoGo task.

### EEG Spectral Power Modulation

**Figure 2:**
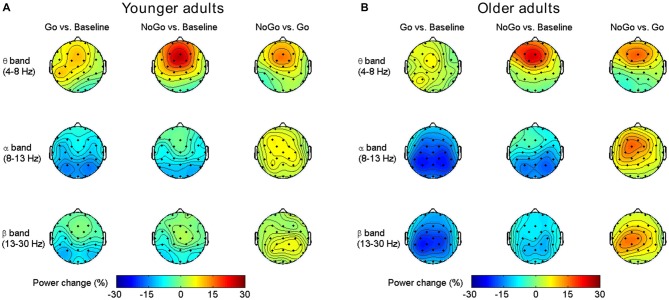
**Group-averaged task-related oscillatory power changes in different frequency bands for the younger group (A) and older group (B)**.

As shown in Figure 2, both younger and older adults showed increased frontal-central theta power during NoGo condition than Baseline condition and Go condition. In alpha and beta band, there were power decreases over central-parietal areas during Go condition than Baseline condition and NoGo condition, and such decreases were stronger in the older group than the younger group. Overall, our results replicated previous studies on EEG band power modulation during Go/NoGo tasks (Leocani et al., 1997; Pfurtscheller and Lopes da Silva, 1999; Kirmizi-Alsan et al., 2006; Huster et al., 2013; Cavanagh and Frank, 2014; Ergen et al., 2014).

### Brain Network Results

#### Small-World Property

**Figure 3:**
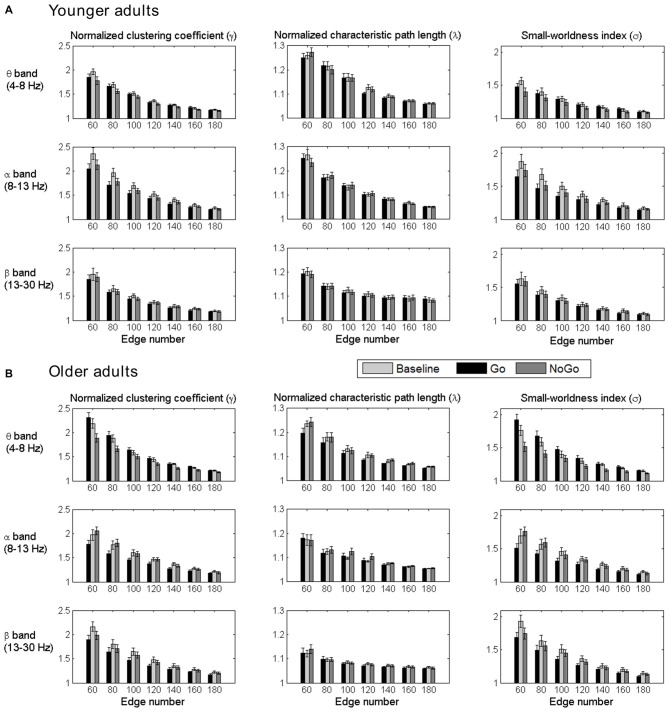
**Group-averaged normalized clustering coefficient (γ), characteristic path length (λ) and small-worldness index (σ) of the brain networks for all task conditions (Baseline, Go, NoGo) in the younger group (A) and older group (B) under different network density levels.** Error bars indicate the standard error of the mean (SEM).

The normalized clustering coefficient (*γ*), characteristic path length (λ) and small-worldness index (*σ*) for younger and older adults within the theta, alpha and beta frequency bands are illustrated in Figure 3. Statistical significance was tested by two-way repeated-measures ANOVA with Task (Baseline, Go and NoGo) as a within-group factor and Age (younger, older) as a between-group factor. The statistical results for *γ*, λ and *σ* are included in Tables 1–3, respectively. The major findings include:

1. Theta band: Main effect of Task was observed for *γ* (*p* < 0.05 under all 7 network density levels), λ (*p* < 0.05 under 3 network density levels) and *σ* (*p* < 0.05 under all 7 network density levels). Follow-up analysis suggested that both age groups showed larger *γ* (*p* < 0.05 under all 7 network density levels), smaller λ (*p* < 0.05 under 5 network density levels) and larger *σ* (*p* < 0.05 under all 7 network density levels) during Go condition than NoGo condition. Furthermore, the difference in *σ* between Go condition and NoGo condition was larger in older adults than that in younger adults, as indicated by significant interaction between Task (Go, NoGo) and Age (younger, older; *p* < 0.05 under 4 network density levels). In addition, older adults showed larger *γ* and *σ* than younger adults during both Go and NoGo conditions (*p* < 0.05 under all 7 network density levels).
2. Alpha band: Main effect of Task was observed for *γ* (*p* < 0.05 under all 7 network density levels) and *σ* (*p* < 0.05 under all 7 network density levels). Follow-up analysis suggested that both age groups showed smaller *γ* (*p* < 0.05 under 4 network density levels) and *σ* (*p* < 0.05 under 3 network density levels) during Go condition than NoGo condition. However, there were no stable main effects or interactions related to Age under different network density levels in the alpha band.
3. Beta band: Main effect of Task was observed for *γ* (*p* < 0.05 under all 7 network density levels) and *σ* (*p* < 0.05 under all 7 network density levels). Follow-up analysis suggested that both age groups showed smaller *γ* (*p* < 0.05 under all 7 network density levels) and smaller *σ* (significant under 6 network density levels) during Go than NoGo condition. Furthermore, older adults showed smaller λ than younger adults during both Go and NoGo conditions (*p* < 0.05 under all 7 network density levels).

**Table 1:** **Results of two-way repeated-measures ANOVA (Task: Baseline vs. Go vs. NoGo; Age: younger vs. older) on normalized clustering coefficient (observed/random)**.

		Factors		
Frequency band	Edge number	Task	Age	Task × Age
Theta (4–8 Hz)	60	***F* = 10.019, *p* < 0.001**	***F* = 8.262, *p* = 0.007**	***F* = 4.057, *p* = 0.021**
	80	***F* = 11.458, *p* < 0.001**	***F* = 8.367, *p* = 0.006**	*F* = 2.045, *p* = 0.136
	100	***F* = 4.609, *p* = 0.013**	***F* = 4.258, *p* = 0.046**	*F* = 0.966, *p* =0.385
	120	***F* = 8.959, *p* < 0.001**	***F* = 7.485, *p* = 0.009**	*F* = 1.565, *p* = 0.216
	140	***F* = 11.839, *p* < 0.001**	***F* = 6.873, *p* = 0.013**	*F* = 1.198, *p* = 0.307
	160	***F* = 10.436, *p* < 0.001**	***F* = 11.981, *p* = 0.001**	*F* = 0.316, *p* = 0.730
	180	***F* = 5.444, *p* = 0.006**	***F* = 12.504, *p* = 0.001**	*F* = 0.878, *p* = 0.420
Alpha (8–13 Hz)	60	***F* = 6.712, *p* = 0.002**	*F* = 3.883, *p* = 0.056	*F* = 2.640, *p* = 0.078
	80	***F* = 9.112, *p* < 0.001**	*F* = 2.568, *p* = 0.083	*F* = 1.182, *p* = 0.284
	100	***F* = 7.107, *p* = 0.003**	*F* = 0.940, *p* = 0.339	*F* = 0.537, *p* = 0.587
	120	***F* = 4.966, *p* = 0.013**	*F* = 0.361, *p* = 0.551	*F* = 1.402, *p* = 0.252
	140	***F* = 10.138, *p* < 0.001**	*F* = 0.832, *p* = 0.367	*F* = 0.267, *p* = 0.767
	160	***F* = 7.474, *p* = 0.001**	*F* = 0.225, *p* = 0.638	*F* = 0.185, *p* = 0.831
	180	***F* = 5.794, *p* = 0.007**	*F* = 0.671, *p* = 0.418	*F* = 0.058, *p* = 0.944
Beta (13–30 Hz)	60	***F* = 9.988, *p* < 0.001**	*F* = 0.702, *p* = 0.407	*F* = 2.320, *p* = 0.105
	80	***F* = 9.379, *p* = 0.001**	*F* = 1.363 *p* = 0.250	*F* = 1.129, *p* = 0.329
	100	***F* = 18.344, *p* < 0.001**	*F* = 1.960, *p* = 0.170	***F* = 5.175, *p* = 0.008**
	120	***F* = 14.281, *p* < 0.001**	*F* = 1.449, *p* = 0.236	***F* = 4.024, *p* = 0.022**
	140	***F* = 14.111, *p* < 0.001**	*F* = 1.062, *p* = 0.309	*F* = 1.502, *p* = 0.229
	160	***F* = 23.374, *p* < 0.001**	*F* = 0.588, *p* = 0.448	*F* = 1.599, *p* = 0.209
	180	***F* = 16.803, *p* < 0.001**	*F* = 0.179, *p* = 0.675	***F* = 5.812, *p* = 0.004**

*Significant results are marked as bold*.

**Table 2:** **Results of two-way repeated-measures ANOVA (Task: Baseline vs. Go vs. NoGo; Age: younger vs. older) on normalized characteristic path length (observed/random)**.

		Factors		
Frequency band	Edge number	Task	Age	Task × Age
Theta (4–8 Hz)	60	***F* = 3.390, *p* = 0.039**	*F* = 3.583, *p* = 0.066	*F* = 0.746, *p* = 0.478
	80	*F* = 0.435, *p* = 0.649	*F* = 3.324, *p* = 0.076	*F* = 1.175, *p* = 0.314
	100	*F* = 0.472, *p* = 0.626	***F* = 6.299, *p* = 0.016**	*F* = 0.261, *p* = 0.771
	120	***F* = 6.260, *p* = 0.003**	*F* = 3.153, *p* = 0.084	*F* = 0.195, *p* = 0.823
	140	*F* = 3.030, *p* = 0.071	*F* = 2.075, *p* = 0.158	*F* = 0.757, *p* = 0.472
	160	*F* = 2.570, *p* = 0.095	*F* = 0.803, *p* = 0.376	*F* = 1.145, *p* = 0.324
	180	***F* = 7.533, *p* = 0.002**	*F* = 0.944, *p* = 0.337	*F* = 1.041, *p* = 0.358
Alpha(8-13 Hz)	60	*F* = 0.510, *p* = 0.602	***F* = 13.099, *p* = 0.001**	*F* = 0.404, *p* = 0.669
	80	*F* = 0.874, *p* = 0.421	***F* = 10.463, *p* = 0.003**	*F* = 0.038, *p* = 0.962
	100	*F* = 3.224, *p* = 0.059	*F* = 4.034, *p* = 0.052	*F* = 0.886, *p* = 0.417
	120	*F* = 2.228, *p* = 0.115	*F* = 1.567, *p* = 0.218	*F* = 0.889, *p* = 0.415
	140	*F* = 0.241, *p* = 0.692	*F* = 1.516, *p* = 0.226	*F* = 0.504, *p* = 0.606
	160	*F* = 0.499, *p* = 0.532	*F* = 0.291, *p* = 0.592	*F* = 1.344, *p* = 0.267
	180	*F* = 0.044, *p* = 0.957	*F* = 1.215, *p* = 0.277	*F* = 1.061, *p* = 0.351
Beta (13–30 Hz)	60	*F* = 0.151, *p* = 0.860	***F* = 11.322, *p* = 0.002**	*F* = 0.747, *p* = 0.477
	80	*F* = 0.196, *p* = 0.822	***F* = 9.128, *p* = 0.004**	*F* = 0.014, *p* = 0.986
	100	*F* = 2.905, *p* = 0.061	***F* = 8.689, *p* = 0.005**	*F* = 0.262, *p* = 0.770
	120	*F* = 1.815, *p* = 0.170	***F* = 8.787, *p* = 0.005**	*F* = 0.017, *p* = 0.983
	140	***F* = 3.998, *p* = 0.039**	***F* = 6.597, *p* = 0.014**	*F* = 2.248, *p* = 0.113
	160	*F* = 0.480, *p* = 0.561	***F* = 5.083, *p* = 0.030**	*F* = 2.580, *p* = 0.082
	180	*F* = 0.504, *p* = 0.579	***F* = 4.710, *p* = 0.036**	*F* = 1.525, *p* = 0.224

*Significant results are marked as bold*.

**Table 3:** **Results of two-way repeated-measures ANOVA (Task: Baseline vs. Go vs. NoGo; Age: younger vs. older) on small-worldness index**.

		Factors		
Frequency band	Edge number	Task	Age	Task × Age
Theta (4–8 Hz)	60	***F* = 13.507, *p* < 0.001**	***F* = 13.344, *p* = 0.001**	***F* = 5.809, *p* = 0.004**
	80	***F* = 9.625, *p* < 0.001**	***F* = 10.069, *p* = 0.003**	***F* = 3.175, *p* = 0.047**
	100	***F* = 4.505, *p* = 0.014**	***F* = 6.705, *p* = 0.014**	*F* = 1.215, *p* = 0.302
	120	***F* = 9.402, *p* < 0.001**	***F* = 7.839, *p* = 0.008**	*F* = 1.403, *p* = 0.252
	140	***F* = 12.770, *p* < 0.001**	***F* = 7.145, *p* = 0.011**	*F* = 1.741, *P* = 0.182
	160	***F* = 11.397, *p* < 0.001**	***F* = 10.748, *p* = 0.002**	*F* = 0.540, *p* = 0.585
	180	***F* = 5.956, *p* = 0.004**	***F* = 12.059, *p* = 0.001**	*F* = 1.101, *p* = 0.338
Alpha (8–13 Hz)	60	***F* = 5.778, *p* = 0.005**	*F* = 0.787, *p* = 0.381	*F* = 1.460, *p* = 0.239
	80	***F* = 7.094, *p* = 0.003**	*F* = 0.069, *p* = 0.794	*F* = 2.152, *p* = 0.123
	100	***F* = 7.320, *p* = 0.002**	*F* = 0.140, *p* = 0.711	*F* = 0.253, *p* = 0.777
	120	***F* = 4.774, *p* = 0.015**	*F* = 0.068, *p* = 0.796	*F* = 0.823, *p* = 0.443
	140	***F* = 9.117, *p* = 0.001**	*F* = 0.357, *p* = 0.554	*F* = 0.146, *p* = 0.865
	160	***F* = 6.219, *p* = 0.003**	*F* = 0.135, *p* = 0.716	*F* = 0.048, *p* = 0.954
	180	***F* = 5.470, *p* = 0.010**	*F* = 0.874, *p* = 0.356	*F* = 0.069, *p* = 0.934
Beta (13–30 Hz)	60	***F* = 9.399, *p* < 0.001**	*F* = 3.040, *p* = 0.089	*F* = 2.955, *p* = 0.058
	80	***F* = 10.485, *p* < 0.001**	*F* = 3.289, *p* = 0.078	*F* = 1.314, *p* = 0.275
	100	***F* = 15.622, *p* < 0.001**	***F* = 4.638, *p* = 0.038**	***F* = 5.972, *p* = 0.004**
	120	***F* = 10.008, *p* < 0.001**	*F* = 4.010, *p* = 0.052	***F* = 3.300, *p* = 0.042**
	140	***F* = 11.703, *p* < 0.001**	*F* = 3.106, *p* = 0.086	*F* = 0.900, *p* = 0.411
	160	***F* = 20.484, *p* < 0.001**	*F* = 2.518, *p* = 0.121	*F* = 0.520, *p* = 0.597
	180	***F* = 13.819, *p* < 0.001**	*F* = 2.815, *p* = 0.066	*F* = 1.714, *p* = 0.198

*Significant results are marked as bold*.

In summary, both younger and older adults showed stable task-related modulations of functional brain networks in theta, alpha and beta bands during the Go/NoGo task. Furthermore, age-related differences were observed in both theta and beta band brain networks. Older adults showed stronger task-related modulations of theta band brain networks than younger adults. In alpha band, however, no stable age-related differences were observed between the two age groups.

#### Node Degree Distribution

**Figure 4:**
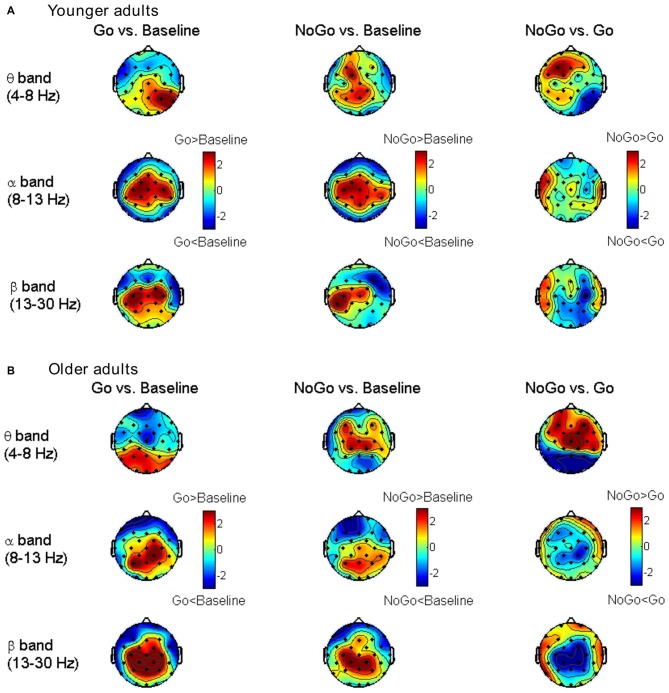
**Topographic maps for *t*-values of the node degree between different task conditions for the younger group (A) and older group (B) in different frequency bands.** The results are illustrated under the network density level of 120 edges.

The differences in node degree between different task conditions (Baseline, Go, NoGo) were tested by paired-samples *t*-test in each age group separately. The statistical *t*-maps under the edge number of 120 (31.75% network density) are presented in Figure 4. Consistent with the small-world property, task-related modulations on node degree could also be clearly observed in theta, alpha and beta bands. In theta band, the frontal-central nodes showed an increase of degree during NoGo condition than Go condition. In beta band, higher node degree was observed during Go condition than NoGo condition among the central-parietal nodes. While in alpha band, such difference between Go condition and NoGo condition was much smaller than that in theta and beta bands.

**Figure 5:**
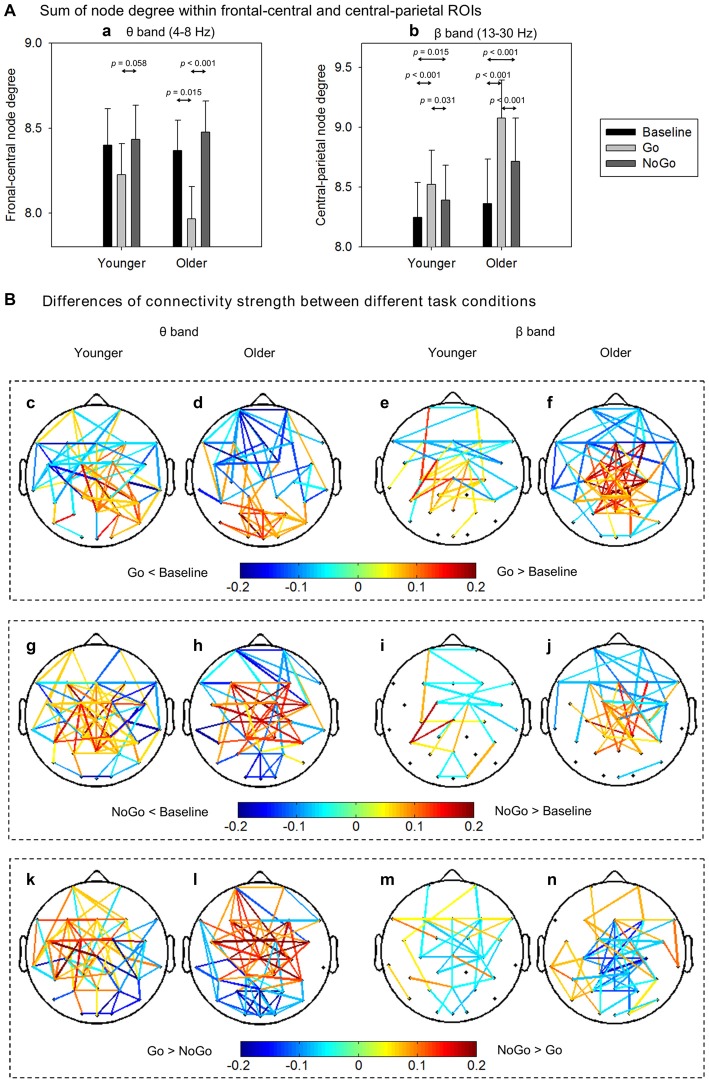
**(A)** Group-averaged node degree within the frontal-central (theta band) and central-parietal (beta band) regions of interest (ROIs). Error bars indicate SEM. **(B)** Group-averaged differences of connectivity strength between different task conditions. Only the connections with absolute differences greater than 0.05 are shown in the figure. The results are illustrated under the network density level of 120 edges.

To quantitatively analyze the task- and age-related effects on node degree in theta and beta band brain networks, we defined two regions of interest (ROIs) based on the *t*-maps in Figure 4: (1) the frontal-central ROI (channels Fp1, Fp2, F3, F4, C3, C4, F7, F8, Fz, Cz, FC1, FC2, FC5, FC6); and (2) the central-parietal ROI (channels C3, C4, P3, P4, Cz, Pz, CP1, CP2, CP5, CP6). The node degree within each ROI was averaged in theta and beta bands, respectively. The ROI-averaged node degree was then subject to a three-way ANOVA with Task (Baseline, Go, NoGo) as a within-group factor and Age (younger, older) as a between-group factor (Figure 5A). In theta band, we observed a main effect of Task (*F*_(2, 76)_ = 9.736, *p* < 0.001) on frontal-central node degree. Follow-up analysis suggested that for younger adults, the task-modulated effects on frontal-central node degree were marginally significant (Go vs. NoGo:* t*_(22)_ = −2.004, *p* = 0.058). For the older adults, in contrast, such task-modulated effects were highly significant (Go vs. Baseline: *t*_(16)_ = −2.725, *p* = 0.015; Go vs. NoGo: *t*_(16)_ = −5.174, *p* < 0.001). In beta band, there was a main effect of Task (*F*_(2, 76)_ = 52.488, *p* < 0.001) and a significant interaction of GNG × Age (*F*_(2, 76)_ = 10.217, *p* < 0.001) on the central-parietal node degree. Follow-up analysis suggested that the task-modulated effects on central-parietal node degree were significant in either the younger (Go vs. Baseline: *t*_(22)_ = 4.604, *p* < 0.001; NoGo vs. Baseline: *t*_(22)_ = 2.632, *p* = 0.015; Go vs. NoGo: *t*_(22)_ = 2.307, *p* = 0.031) or the older (Go vs. Baseline: *t*_(16)_ = 7.674, *p* < 0.001; NoGo vs. Baseline: *t*_(16)_ = 4.404, *p* < 0.001; Go vs. NoGo:* t*_(16)_ = 5.205, *p* < 0.001) group.

To further characterize the age-related differences in task-modulated effects on node degree, we calculated the relative changes of ROI-averaged node degree between Go condition and NoGo condition, and then compared the relative changes between the two groups using independent-samples *t*-test. Older adults showed significantly larger relative changes of node degree than younger adults in both the theta (frontal-central ROI, *t*_(38)_ = 2.228, *p* = 0.032) and the beta (central-parietal ROI, *t*_(38)_ = −2.540, *p* = 0.015) bands. Figure 5B illustrates the main connections that have different strengths between different task conditions. Apparently, in theta band brain network, NoGo condition evoked stronger cortical connections than Go condition within frontal-central area (Figures 5Bk,l), while in beta band brain network, Go condition evoked stronger cortical connections than NoGo condition within central-parietal area (Figures 5Bm,n).

## Discussion

We present a thorough analysis on the task-related modulations on induced EEG activities as well as the effects caused by normal aging during a Go/NoGo task. Behaviorally, both younger and older adults showed high accuracy and negligible FARs in the Go/NoGo task, suggesting a sufficient level of inhibitory performance. Fourier analysis revealed an increase in frontal-central theta power during NoGo condition, and decreased central-parietal alpha and beta power during Go condition, which replicated previous findings in the literature. For the graph theoretical analysis on oscillatory brain networks, both age groups showed classic small-world brain networks in theta, alpha and beta bands, and older adults showed stronger task-modulated effects on small-world property than younger adults. Meanwhile, such frequency-specific modulation of brain networks were spatially segregated, indicating the differences of brain network between response inhibition and response execution. Specifically, theta-band brain network showed larger frontal-central node degree in NoGo condition than that in Go condition, whereas beta-band brain network showed larger central-parietal node degree in Go condition than that in NoGo condition. Moreover, these task-related modulations on node degree were also stronger in older adults than younger adults. Taken together, our findings indicate that the topological organization of oscillatory brain networks in theta and beta bands might serve as a hallmark for response inhibition and execution, which might become stronger and more robust due to normal aging.

### Task-Related Effects on Oscillatory Brain Networks

It is commonly agreed that small-worldness implies both high local clustering and short path length, which reflects an optimal balance between local segregation and global integration of brain networks (Watts and Strogatz, 1998; Bassett and Bullmore, 2006; Rubinov and Sporns, 2010). Several studies have documented the small-world organization of oscillatory brain networks in simple motor tasks, i.e., finger or foot movements, and resting state (Bassett et al., 2006; De Vico Fallani et al., 2008; Jin et al., 2012). In this study, we further demonstrated the small-worldness of brain networks in a Go/NoGo task that requires high-level cognitive computations. More importantly, we found that the index of small-worldness (*σ*) was significantly different between Go condition and NoGo condition. Although previous research failed to observe significantly different small-worldness index between simple motor tasks and the resting state, there seemed to be an overall trend of decreasing small-worldness indices during a finger-tapping task compared with resting state in the beta band network (Jin et al., 2012). These results were in line with our findings of the decreased small-worldness index in Go condition than Baseline condition and NoGo condition in beta band network. In theta band, however, we observed an increase of small-worldness index in Go condition than NoGo condition. These findings indicate that the theta and beta band brain networks play different functional roles in the Go/NoGo task, which is concordant with the literature, that is, the theta-band phase synchrony is more likely to be involved in inhibitory process (NoGo; Brier et al., 2010; Muller and Anokhin, 2012; Anguera et al., 2013b), while the beta-band phase synchrony plays a major role in motor production (Go; Aoki et al., 2001; Brovelli et al., 2004; Jin et al., 2012).

The quantitative analysis of node degree further supported that theta- and beta-band phase synchrony played different functional roles in the Go/NoGo task. In theta band brain network, response inhibition significantly enhanced the frontal-central node degree (Figure 5A). This finding coincides well with the current understanding of theta oscillations, that is, frontal theta phase synchrony is commonly enhanced when more cognitive control is required (Cavanagh and Frank, 2014). On the other hand, in beta band brain network, response execution significantly enhanced the central-parietal node degree (Figure 5B), indicating that motor response is associated with the increase of beta-band synchrony which enhances cortical connections with or within the sensorimotor areas (Mima et al., 2000; Bassett et al., 2006; Jin et al., 2012).

In theta band network, task-modulated effects on small-worldness manifested in significantly decreased clustering coefficient and increased characteristic path length in NoGo condition than that in Go condition. In the beta band network, however, the task-modulated effects on small-worldness index was only presented in the significant changes in clustering coefficient (Figure 3). Given that clustering coefficient and characteristic path length represent local segregation and global integration of complex networks, respectively (Watts and Strogatz, 1998; Rubinov and Sporns, 2010; Bullmore and Bassett, 2011), our findings suggest that theta band brain network involved more distant cortical connections than beta band brain network. Furthermore, this inference was also supported by the differences in connectivity strength between Go condition and NoGo condition. Specifically, task-related changes (NoGo > Go) in theta band brain network involved relatively large-scale cortical connections, including the frontal, central and parietal areas (Figures 5Bk,l), whereas in beta band brain network, task-related changes (Go > NoGo) in cortical connections were primarily concentrated around the sensorimotor area (Figures 5Bm,n).

### Aging Effects on Oscillatory Brain Networks

Compared with younger adults, the small-worldness as well as task-modulated effects were well preserved in older adults. Furthermore, the task-modulated effects on node degree distribution in theta and beta band brain network were more prominent in older adults than younger adults (Figure 5A). There have been neuroimaging evidences that older adults could recruit more frontal activation than younger adults in cognitive control tasks, i.e., the Go/NoGo task, reflecting a functional compensation (Rajah and D’Esposito, 2005; Park and Reuter-Lorenz, 2009; Spreng et al., 2010; Heilbronner and Münte, 2013; Hong et al., 2014). Therefore, it could be inferred that normal aging not only increases the functional activation in specific regions, but also enhances the brain functional connections, which might indicate the recruitment of additional resources, and such findings are consistent with recent functional connectivity study based on functional magnetic resonance imaging (fMRI; Geerligs et al., 2014). Collectively, our findings clearly show that normal aging does not reduce, but rather enhances the neural synchrony during cognitively demanding tasks, which could shed new light on the neural mechanisms of cognitive aging when combined with the previously reported decrease in neural synchrony due to pathological aging (Pijnenburg et al., 2004; Uhlhaas and Singer, 2006; Stam et al., 2007, 2009; Knyazeva et al., 2010).

An attention-cueing Go/NoGo task with cue-target design, rather than a simple Go/NoGo task was used in this study. In such cue-target paradigm, Go/NoGo stimuli were always preceded by an instructive cue that led to increased response preparation in order to get a fast response to Go-stimulus. In this case, a prepared response had to be aborted when a NoGo-stimulus appeared at the cued location, which led to a robust response inhibition process (Bruin et al., 2001; Smith et al., 2006, 2007). Consistently, significant inhibition-related ERP components had been reported in our previous study (Hong et al., 2014). Moreover, since this study focused on response inhibition and execution, attention-related cognitive process and brain activity was not included here, which though, had been reported elsewhere (Hong et al., 2015).

In this study, the averaged ERP activity was subtracted from EEG signals before PS analysis to eliminate the effects from evoked activities that are phase-locked to the stimulus onset, i.e., N2 and P3 components. The N2 and P3 components have been widely reported to be the neural marker of response inhibition (Falkenstein et al., 1999; Albert et al., 2013; Huster et al., 2013; Hong et al., 2014). Since the ERP waves have subtracted before phase synchrony and brain network analysis, our findings suggest that task-modulated brain network constructed from induced (non-phase-locked) EEG activity could serve as another possible neural marker that is independent of conventional ERP markers. Moreover, such marker of brain network could be well preserved and even become stronger during normal aging.

One limitation in this study should be noted. Following a common approach in the literature (Dietl et al., 1999; Doppelmayr et al., 2000; Gruber et al., 2002; Deiber et al., 2009), we subtracted the averaged ERP from EEG epochs to eliminate the effects from evoked activity in this study. Such approach is based on the assumption that the same ERP is present in each single trial, which however, may be problematic. Unfortunately, extracting precise ERP activity at single trial level is still a highly challenging task, and currently there is still lack of widely accepted method in this field. Future work is required to address this limitation.

To conclude, by employing graph theoretical analysis, we thoroughly investigated the age-related differences in synchronous neural network within functionally-distinct frequency bands in a Go/NoGo task. This study explicitly demonstrated a close relationship between the frequency-specific neural synchrony and response inhibition as well as response execution. Our findings could also provide important implications into the current understanding of the neural mechanisms of cognitive aging from the perspective of synchronous brain networks.

## Author Contributions

XH, YL, JS and ST designed research; XH performed research; XH, YL and JS analyzed data; XH, YL and ST wrote the article.

## Conflict of Interest Statement

The authors declare that the research was conducted in the absence of any commercial or financial relationships that could be construed as a potential conflict of interest.
